# Decreased Volume of Lateral and Medial Geniculate Nuclei in Patients with LHON Disease—7 Tesla MRI Study

**DOI:** 10.3390/jcm9092914

**Published:** 2020-09-10

**Authors:** Kamil Jonak, Paweł Krukow, Katarzyna E. Jonak, Elżbieta Radzikowska, Jacek Baj, Anna Niedziałek, Anna Pankowska, Mark Symms, Andrzej Stępniewski, Arkadiusz Podkowiński, Ida Osuchowska, Cezary Grochowski

**Affiliations:** 1 Department of Clinical Neuropsychiatry, Medical University of Lublin, 20-439 Lublin, Poland; jonak.kamil@gmail.com (K.J.); pawelkrukow@umlub.pl (P.K.); 2 Department of Biomedical Engineering, Lublin University of Technology, 20-618 Lublin, Poland; 3 Department of Foreign Languages, Medical University of Lublin, Jaczewskiego 4, 20-090 Lublin, Poland; kasia.jonak@gmail.com; 4 Department of Plastic Surgery, Central Clinical Hospital of the MSWiA in Warsaw, 01-211 Warsaw, Poland; elzbieta.radzikowska@gmail.com; 5 Department of Anatomy, Medical University of Lublin, 20-400 Lublin, Poland; jacek.baj@me.com; 6 Department of Radiography, Medical University of Lublin, 20-081 Lublin, Poland; Anna.niedzialek@umlub.pl (A.N.); anna.pankowska@umlub.pl (A.P.); 7 General Electric (GE) Healthcare, Amersham Place, Amersham HP7 9NA, UK; MarkRoger.Symms@ge.com; 8 Ecotech-Complex, Maria Curie-Skłodowska University, 20-612 Lublin, Poland; andrzej.stepniewski@poczta.umcs.lublin.pl; 9 Specialized Neurosurgical Practice, 20-092 Lublin, Poland; apodkowinski@wp.pl; 10 Laboratory of Virtual Man, Chair of Anatomy, Medical University of Lublin, 20-400 Lublin, Poland; i.m.osuchowska@gmail.com

**Keywords:** LHON, LGN, MGN, mitochondrial

## Abstract

Leber’s hereditary optic neuropathy (LHON) is a maternally inherited genetic disorder leading to severe and bilateral loss of central vision, with a young male predilection. In recent years, multiple studies examined structural abnormalities in visual white matter tracts such as the optic tract and optic radiation. However, it is still unclear if the disease alters only some parts of the white matter architecture or whether the changes also affect grey matter parts of the visual pathway. This study aimed at improving our understanding of morphometric changes in the lateral (LGN) and medial (MGN) geniculate nuclei and their associations with the clinical picture in LHON by the application of a submillimeter surface-based analysis approach to the ultra-high-field 7T magnetic resonance imaging data. To meet these goals, fifteen LHON patients and fifteen age-matched healthy subjects were examined. A quantitative analysis of the LGN and MGN volume was performed for all individuals. Additionally, morphometric results of LGN and MGN were correlated with variables covering selected aspects of the clinical picture of LHON. In comparison with healthy controls (HC), LHON participants showed a significantly decreased volume of the right LGN and the right MGN. Nevertheless, the volume of the right LGN was strongly correlated with the averaged thickness value of the right retinal nerve fiber layer (RNFL). The abnormalities in the volume of the LHON patients’ thalamic nuclei indicate that the disease can cause changes not only in the white matter areas constituting visual tracts but also in the grey matter structures. Furthermore, the correlation between the changes in the LGN volume and the RNFL, as well as the right optic nerve surface area located proximally to the eyeball, suggest some associations between the atrophy of these structures. However, to fully confirm this observation, longitudinal studies should be conducted.

## 1. Introduction

Leber’s hereditary optic neuropathy (LHON) is described as a maternally inherited genetic disorder, with a young male predilection and loss of central vision, which is almost always bilateral and severe [1,2]. Over 90% of LHON cases have one of the three mitochondrial mutations (mtDNA): m.3460G> A (MTND1), m.11778G> A (MTND4) and m.14484T> C (MTND6) [3,4]. All of these three primary mtDNA mutations disturb the flux of electrons along the mitochondrial respiratory chain, which leads to impaired oxidative phosphorylation (OXPHOS) and increased levels of reactive oxygen species (ROS) [5]. Researchers have also observed some pathological changes, such as retinal ganglion cell degeneration with axonal loss of the optic nerve and thickening of the retinal nerve fiber layer (RNFL), in LHON patients [6]. Additionally, these pathological changes result in a decline in visual acuity (VA), permanent central scotoma and, in the end, optic nerve atrophy.

The thalamus is one of the most important subcortical brain structures that plays a key role in information transfer between different subcortical areas and the cerebral cortex [7]. Because of its location, the thalamus also contributes significantly to diseases connected with vision loss [8]. Lateral geniculate nucleus (LGN) is a structure located in the metathalamus and is responsible for the connectivity between the optic nerve and the primary visual cortex. The LGN, through the optic tract, receives the information from the retinal ganglion cells as well as the reticular activating system. Through optic radiation, the signal leaves the LGN and ends in the primary visual cortex. Moreover, a great number of feedback connections run backwards from the primary visual cortex to the LGN [9]. Three cell types were differentiated as components of LGN, namely parvocellular, magnocellular and koniocellular cells. Parvocellular as well as magnocellular cells receive signals from retinal ganglion cells (RGC). Moreover, magnocellular cells receive input from the motion-sensitive Y-type RGC and parvocellular cells receive input from color-sensitive X-type RGC. Koniocellular cells send projections into the ventral regions of the previously mentioned cells. Moreover, several neuronal loops, such as the central bundle, Baum’s loop as well as Meyer’s loop, originate from the LGN and project through the internal capsule to the spiny stellate neurons in the primary visual cortex [10]. Another important thalamic nucleus involved in sensory processing is represented by the medial geniculate nuclei (MGN). The MGN represents the thalamic relay station of the auditory tract and, thus, the gateway to the centers of auditory perception in the cerebral cortex [11]. Together with the lateral geniculate nucleus, which is the relay station of the optic system, it includes the metathalamus. Previous studies showed a task-dependent modulation in the auditory sensory thalamus for auditory speech recognition [12,13], as well as a task-dependent modulation in the visual sensory thalamus for visual speech recognition [12].

The development of novel MR imaging protocols for direct visualization and anatomical delineation of thalamic structures has been increasing in importance over the past few years. Early research by Deoni et al. [14] stated that T1 values ranging from 700 to 1400 ms at 1.5 T show varying contrasts in intrathalamic nuclei differentiation. Modifications of the magnetization-prepared rapidly acquired gradient echo (MPRAGE) sequence were proposed through the use of different inversion times (TI) to visualize boundaries between some thalamic structures through intensity variations caused by a difference in myelin concentration [15,16]. With higher spatial resolution and signal-to-noise ratio (SNR), the accuracy of these sequences acquired at the lower field can be further enhanced thanks to recent progress in high-field imaging. Abosch et al. [17] explored the use of 7T susceptibility weighted imaging (SWI) images, while Tourdias et al. [18] submitted a protocol for an optimal 7T anatomical sequence. Therefore, thanks to higher SNR values, ultra-high-field magnetic resonance imaging allows the visualization of tiny thalamic nuclei such as LGN with high sensitivity, higher contrast and better spatial resolution, in comparison to 3 Tesla MRI [19]. A study performed on glaucoma patients, using 7 Tesla MRI, revealed decreased LGN volume compared to the healthy controls, proving ultra-high-field MRI as a useful tool in diagnosing ophthalmological diseases [20]. Anatomical abnormalities reported in the studies usage of 1.5 T and 3 T MRI’s showed optic nerve atrophy with the increased T2-weighted signal [21,22] and structural damage of the visual cortex and the retinofugal pathway, which could be related to axonal degeneration secondary to the loss of retinal ganglion cells [23,24]. Additionally, a morphometric study showed a reduction of the volume in the optic chiasm, optic tract, optic radiations and primary visual cortex in the LHON patient groups [23]. In this case, visual cortex changes were also significantly correlated with global and temporal peripapillary retinal nerve fiber layer thickness. Despite the mentioned MRI-based studies investigating LHON, it is still unclear how the disease, and its functional consequences associated with blindness, might influence individual subcortical structures—for example, its volume.

The main aim of this study was to evaluate the volume of the lateral and medial geniculate nucleus in patients with LHON disease. As was mentioned, the LGN is an important part of the optic tract in the human brain and, in accordance with earlier studies which have shown multiple changes in several white-matter (WM) areas in LHON, there is a serious supposition that these changes also affect the LGN. Furthermore, in accordance with previous papers that reported hearing problems in LHON participants, we also wanted to evaluate the volume of MGN in LHON participants in comparison to health controls (HC). To achieve these goals, we applied submillimeter high-field MRI for the evaluation of morphometric changes in LHON patients. High precision of 7T MRI could reveal some significant associations between brain anomalies and selected features of the clinical picture of this disease, such as illness duration, averaged RNFL thickness or the optic nerve examination results. Establishing such significant relationships could deepen current knowledge on the progression of the disease and its neuroanatomic basis.

## 2. Methods

### 2.1. Subjects

Initially, twenty-five patients with LHON were selected from the national health database. However, only 15 of them met the final inclusion criteria, which were the following: 11778G>A mitochondrial DNA mutation confirmed by genetic tests, no known pathological changes within the cerebrovascular system, capable of signing informed consent, over 18 years old, at least 10 years of regular education and no family history of severe neuropsychiatric disorders, which would additionally affect the state of the nervous system. Patients did not suffer from hypertension, diabetes or any neurodegenerative diseases. Additionally, patients with any metallic implant, who were pregnant or breastfeeding, as well as those suffering from claustrophobia, were excluded from the study. Radiological assessment was carried out by an experienced neuroradiologist (25 years of experience) and a neuroanatomy specialist (40 years of experience). Participants who received idebenone during the treatment were excluded from the analysis; however, seven of our participants with the shortest duration of illness were about to begin the treatment shortly after our research had been completed. Two of our participants were also related and had a family history of LHON. The HC group was recruited from the local community after the clinical group was completed in order to guarantee the demographic matching of individuals from both samples. All of the participants were right-handed non-smokers with no history of chronic alcohol consumption. Blood pressure was measured in all participants, and no abnormalities were found. All participants signed informed consent. This research was approved by the local medical ethics committee of the Medical University of Lublin (KE-0254/23/2017) and was carried out in compliance with national legislation and the Declaration of Helsinki. The scans were obtained at the Ecotech Complex, Lublin, Poland.

### 2.2. Optical Coherence Tomography (OCT) Acquisition

Retina structural evaluation was performed using optical coherence tomography (OCT; Revo NX 130, Optopol, Poland). Images with blinking artifacts or involuntary saccades and with a signal strength lower than 6 were discarded. After visual inspection, images with segmentation failure were excluded. Each subject was scanned at least three times, and, for future analysis, only the best scans were selected. The OCT software uses an automated computerized algorithm to rank the RNFL thickness against a normal percentile. Scale distribution was derived from a database of age-matched control subjects and designated them into four different categories: normal (5–95th percentile), below normal (<5th percentile), markedly below normal (<1% percentile) or supra-normal (>95th percentile). At the time of the scanning, pupil dilatation was induced in all the subjects, and an internal fixation was used whenever possible. RNFL scan protocol was used with a preset diameter of 3.45 mm and was centered on the optic nerve disk by a trained technologist. For each eye, we measured average peripapillary RNFL thickness (360° measure).

### 2.3. MRI Acquisition

Three-dimensional inversion recovery-prepared spoiled gradient echo (3D-SPGR “BRAVO”) was acquired from the 7T MRI with a 32-channel coil at the Ecotech Complex Lublin. The field of view was 220 × 220 × 180 mm and the acquisition matrix was 256 × 256 × 180. The images were reconstructed to a 512 × 512 matrix, giving a final voxel size of 0.43 × 0.43 × 1 mm. TE 2.6 ms, TR 6.6 ms, TI 450 ms, flip angle 12 degrees, bandwidth ±31.25 kHz. Parallel imaging (ARC) factor 2 was used.

For the evaluation of the optic nerve dimensions in the LHON group, we used “Silent” zero echo time imaging protocol, which was described in detail in our previous work [25]. In brief, data were acquired using the “Silent-MT” sequence. Silent is a three-dimensional version of the sequence RUFIS, first introduced by Madio and Lowe [26]. The fat suppression pulse was applied once every 192 spokes; this pulse has been shown to introduce magnetization transfer contrast to the image. A matrix of 192 × 192 × 192 was acquired over a field of view of 15.4 cm, yielding an isotropic resolution of 0.8 mm. Scan time was 2 min.

### 2.4. Image Analysis

**Figure 1:**
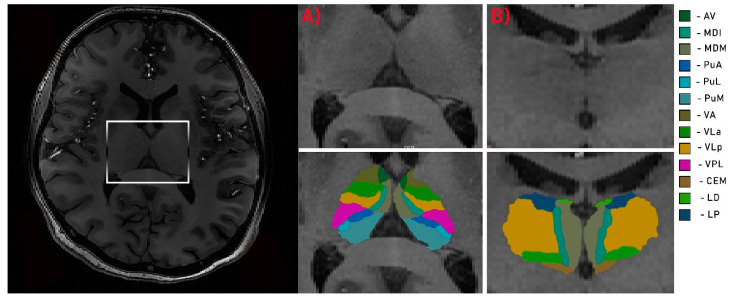
Segmentation of thalamus. Axial (**A**) and coronal (**B**) views of the thalamus. Av—anteroventral; MDI—mediodorsal lateral parvocellular; MDM—mediodorsal medial magnocellular; PuA—pulvinar anterior; PuL—pulvinar lateral; PuM—pulvinar medial; VA—ventral anterior; VLa—ventral lateral anterior; VLp—ventral lateral posterior; VPL—ventral posterolateral; CEM—central medial; LD—laterodorsal; LP—lateral posterior.

**Figure 2:**
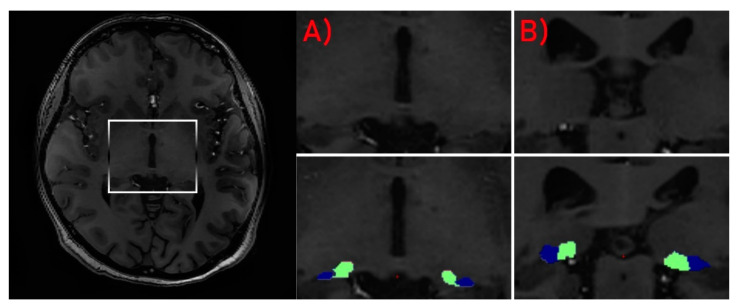
Segmentation of LGN (blue) and MGN (green). Axial (**A**) and coronal (**B**) views of the nuclei.

Due to high-field inhomogeneity in 7T MRI, each structural volume was intensity bias corrected using the unified segmentation process [27] algorithms in SPM 12 (http://www.fil.ion.ucl.ac.uk/spm; MATLAB R2018A version, Mathworks, Inc., Natick, MA, USA). Brain segmentation procedure was performed using the “recon-all” function in the FreeSurfer program (http://surfer.nmr.mgh.harvard.edu/). For stable processing, voxel size was down sampled to 0.5 mm^3^ from the native size. Surface inflation number was set as 100 and implemented into recon-all as –cm flag function [28]. Recon-all image processing procedure, including normalization of signal intensity, skull stripping to separate areas of the skull in the normalized space and automatic segmentation, was performed. After the initial preprocessing, quality assessment was conducted by the radiologist. Each slice was visually inspected for skull stripping errors, segmentation errors, normalization errors, pial surface errors and topological defects following the FreeSurfer guidelines. The appropriate preprocessing steps were then repeated for the participants whose images required editing. In the second step of analysis, individual thalamic nuclei were segmented. We obtained the absolute individual thalamic nuclei volumes from the results of recon-all brain segmentation by the application of a Bayesian segmentation method based on a probabilistic atlas derived from histology (Figure 1 and Figure 2) [29].

**Figure 3:**
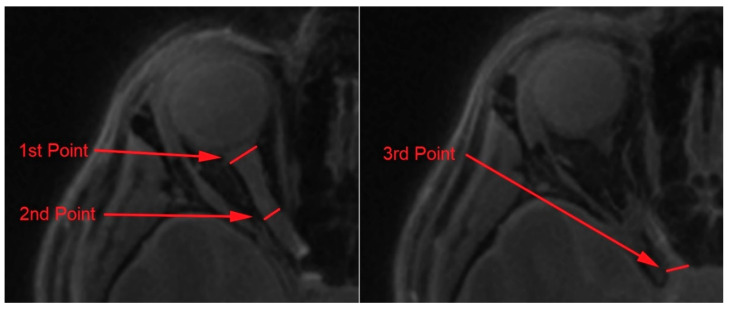
Measurement of the optic nerve dimensions at three selected points: (1) 1st point of interest, (2) 2nd point of interest, (3) 3rd point of interest.

An analysis of the optic nerves’ dimensions was carried out using the OsiriX Lite software (OsiriX); the whole procedure was also described in detail in our previous work [25]. Firstly, the optic nerves were manually segmented from the Silent protocol with the application of Repulsor and region of interest tools. Secondly, to remove the remaining unwanted surrounding tissue, the nerves were reconstructed into a 3D model. Lastly, the nerve length and dimensions were captured in three different points (Figure 3). The selected measurement points were placed as follows: the first point—proximally to the eyeball; the second point—the middle area of the optic nerve; the third point—proximally to the optic chiasm. Nerve dimensions, cross-sectional surface areas and length were calculated by two independent observers (neuroradiologist and neuroanatomy specialist) using the OsiriX built-in standardized functions.

### 2.5. Statistical Analysis

To compare the studied groups in terms of basic demographic variables, a two-sided Student’s t-test was used for quantitative variables and non-parametric χ^2^ for qualitative characteristics. Differences between left and right nerve diameters in the LHON group were calculated with non-parametric Mann–Whitney test (Z) and *p* < 0.05 was set as a statistical significance threshold. Two types of analysis of variance have been implemented to study between- and within-groups effects regarding volumetric data: analysis of covariance (ANCOVA) with age and sex as controlled factors in groups comparison and within-subject ANOVA to analyze whether groups had specific features of assessed structures’ asymmetry (right versus left neural area). Within-group ANOVAs were computed in each group separately, and each computation covered one pair of variables (e.g., volumes of left and right MGN). The level of statistical significance in ANCOVA included correction for multiple testing; in all cases, the effects were considered statistically significant if *p* < 0.05. Partial eta squared (η_p_^2^) was an indicator of effect size. After establishing a set of volumetric variables significantly differentiating the groups, it was correlated with selected clinical (e.g., duration of illness) and ophthalmologic characteristics (e.g., RNFL, optic nerve dimensions), with application of Pearson r-test and FDR (false discovery rate) correction. Correlations were verified only in the LHON sample.

## 3. Results

### 3.1. Participants

Table 1 presents demographic and clinical data on the studied groups. Samples did not differ significantly in terms of age (LHON = 36.21; HC = 32.53), sex (LHON = 86% male; HC = 66% male) or years of education (LHON = 15.33; HC = 16). In the LHON group, the duration of illness was around 10 years and the RNFL averaged thickness was left: 62.153 and right: 62.054. Averaged length of the optic nerve in the LHON group was 4.18 cm for the right and 4.23 cm for the left side. Nevertheless, the analysis of the surface areas and diameters of both nerves showed no significant differences between optic nerves (Table 2).

### 3.2. Between-Group Comparisons

**Figure 4:**
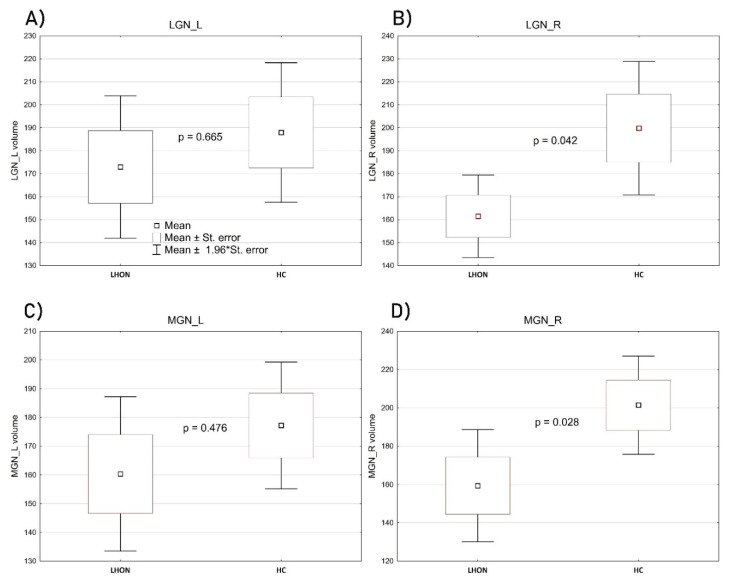
Between-group comparisons of the LGN ((**A**)—left, (**B**)—right) and MGN ((**C**)—left, (**D**)—right) volumes (mm^3^). The figures show mean, standard error, the range of 1.96 standard error and the level of statistical difference based on ANCOVA computations. The widest range of results distribution, as depicted in the chart, covers mean ±1.96 x standard error.

Although the groups (LHON and HC) did not differ significantly in terms of age and sex, due to the specificity of volumetric data, consisting in the necessity to ensure precise measurements of relatively small structures, we conducted the groups’ comparison by controlling for these two demographic characteristics as included covariates. The volume of the left LGN was relatively similar in LHON and in the control sample: F(1, 26) = 0.191, *p* = 0.665, η_p_^2^ = 0.007; however, in this case, age was a significant contributor: F(1, 26) = 4.512, *p* = 0.042, η_p_^2^ = 0.14. The volume of the right LGN was significantly decreased in the LHON sample compared with HC: F(1, 26) = 4.516, *p* = 0.042, η_p_^2^ = 0.14. None of the included covariates had a significant contribution to this comparison. Figure 4B shows the scope of the difference. Analogous results come from samples’ comparison regarding the left and the right medial geniculate nuclei (MGN). Again, the left one did not differentiate the groups: F(1, 26) = 0.522, *p* = 0.476, η_p_^2^ = 0.01. Age was a significant contributor: F(1, 26) = 4.706, *p* = 0.039, η_p_^2^ = 0.15, while the volume of the right MGN was significantly lower in LHON patients than in controls: F(1, 26) = 5.350, *p* = 0.028, η_p_^2^ = 0.16 (Figure 4D). An inter-group comparison of the right MGN was not affected by any of the controlled covariates.

### 3.3. Within-Group Effects

**Figure 5:**
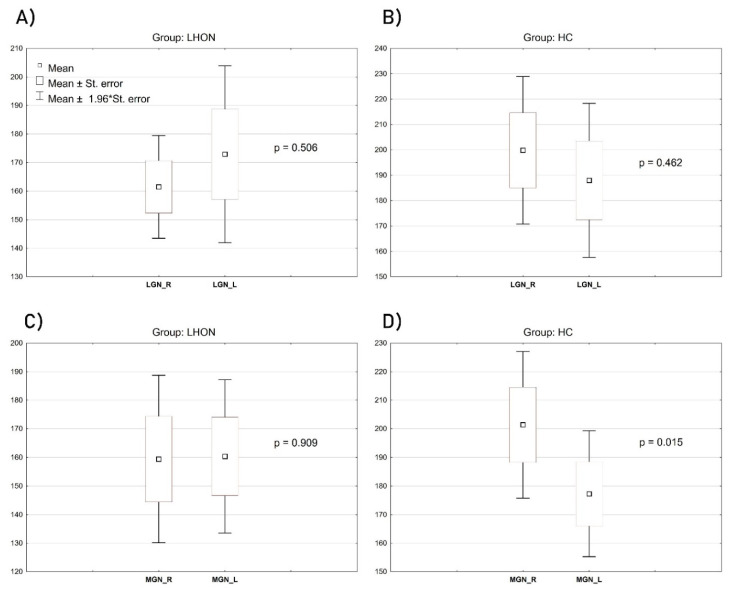
Within-group effects of the right and left LGN (**A**,**B**) and the right and left MGN (**C**,**D**) (mm^3^). The figures show mean, standard error, the range of 1.96 standard error and the level of statistical significance of the within-subjects effect.

To verify whether the groups revealed specific features of the assessed structures’ asymmetry (e.g., right versus left LGN), a set of within-subjects ANOVAs was performed separately in each of the studied samples (Figure 5). There were no significant within-subjects effects regarding the left and the right LGN in the LHON group: F(1, 14) = 0.465, *p* = 0.506, η_p_^2^ = 0.03, or in the HC group: F(1, 14) = 0.569, *p* = 0.462, η_p_^2^ = 0.03. In LHON patients, there was also no significant asymmetry regarding the left and the right MGN: F(1, 14) = 0.013, *p* = 0.909, η_p_^2^ < 0.01. However, such an asymmetry was noticed in controls: F(1, 14) = 7.584, *p* = 0.015, η_p_^2^ = 0.35, with the volume of the right MGN significantly larger than the left (Figure 5D).

### 3.4. Volumetric—Clinical Correlations in LHON Sample

**Figure 6:**
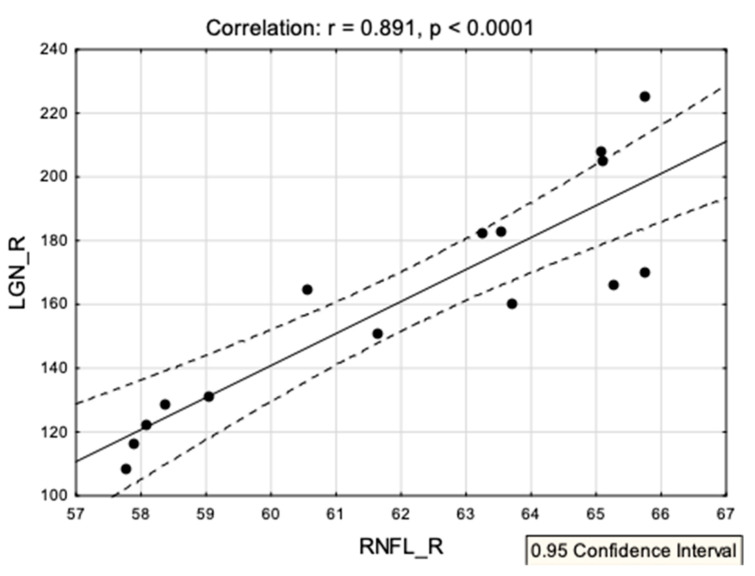
A correlation scatterplot showing associations between the volume of the right LGN and the right retinal nerve fiber layer in the LHON group.

**Figure 7:**
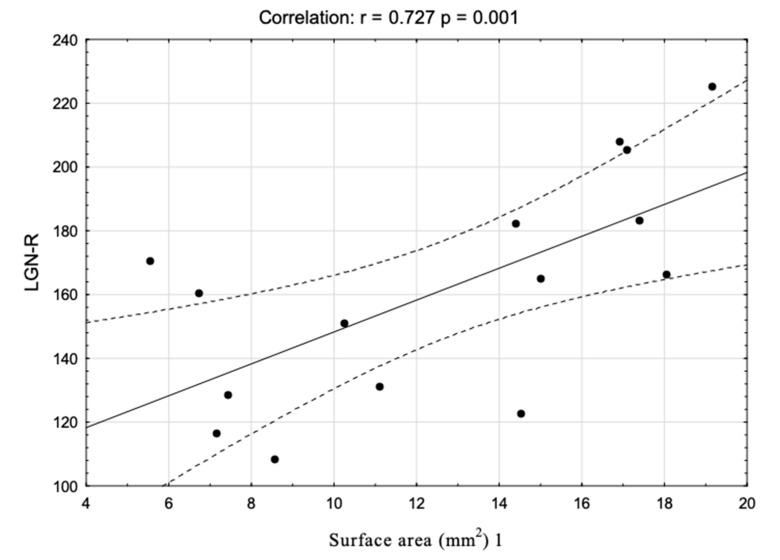
A correlation scatterplot showing associations between the volume of the right LGN and right optic nerve surface area 1 in the LHON group.

Among all the variables significantly differentiating the groups’ clinical and ophthalmologic characteristics, only the correlation between the volume of the right LGN and the right retinal nerve fiber layer survived the FDR correction: r = 0.89, *p* < 0.0001. Figure 6 shows the correlations’ scatterplot. Additionally, we have analyzed the hypothetical connection between the LGN volume (right and left) and the different diameters of the optic nerves. Only one significant correlation survived the FDR correction: between the right nerve surface area 1 and the right LGN volume (Figure 7).

## 4. Discussion

The main goal of this study was to investigate changes in the volume of the lateral and medial geniculate nucleus in LHON patients. For this purpose, we applied submillimeter ultra-high-field MRI morphometry to the thalamic nuclei segmentation, which made it possible to perform precise delineation of the LGN and MGN areas. Moreover, we have demonstrated that abnormalities within these structures were associated with selected features of the disease’s clinical picture. The most important findings of this study were that, in comparison to healthy controls, LHON patients showed significantly decreased volume of the right LGN and the right MGN. Furthermore, we have also found that the volume of the right LGN was significantly correlated with the averaged thickness of the right RNFL in the LHON participants. The analysis of the optic nerve did not show any significant differences between the right and the left optic nerve in any of the measured points. However, we have observed one significant correlation between the volume of right LGN and the right optic nerve surface area in the first measurement point. The volume comparison within the groups did not show any significant results in LGN and MGN asymmetry in the LHON group. Additionally, we found asymmetry between the left and right MGN in controls.

The lateral geniculate nucleus is a structure located in the metathalamus which acts as a transmitter of the visual pathway, providing an interconnection of the optic nerve as well as the optic tract and optic radiation. The results of this study showed that the volume of the right LGN was significantly decreased in the LHON sample compared with HC. Such findings were described in normal tension glaucoma patients and were analyzed by 7 Tesla MRI as well. The LGN volume was significantly decreased in these patients compared to controls. Moreover, axonal degeneration of the optic tract and optic radiation was confirmed by the diffusion tensor imaging (DTI) [20]. A study carried out by Rizzo et al. [30] analyzed 22 LHON patients using 1.5 Tesla MRI to assess the changes in the optic-radiation and, additionally, analysis was expanded to include post-mortem pathologic examination of two patients. They did not report any abnormalities in LHON patients and mutation carriers compared to the control group. However, the post-mortem examination of the LGNs harvested from the LHON patients showed a decrease in the average neuron soma across all six layers. The average neuron density of the LHON LGN was decreased across all layers and the changes were consistent across all the layers of the LGN as the percent decrease in density was the same for both magnocellular and parvocellular layers, with a similar ratio between the magnocellular and parvocellular layers either in the LHON or the controls. The results obtained using 7 Tesla MRI were consistent with the post-mortem findings in the study performed by Rizzo et al. Moreover, in 10 out of 15 patients analyzed in this study, the disease firstly affected the right optic nerve and the results of the ophthalmological examination provided by the patients indicated the disease to be more severe at the right eye. The images showed significant atrophy of the right LGN in LHON patients.

In the literature, several papers can be found which report the co-existence of LHON and hearing loss [31,32,33]. Yang et al. created a database including 26,000 mitochondrial genomes, in which more than 200 individuals were found to have a co-occurrence of pathogenic mutations for LHON and hearing loss [34]. The MGN is a part of the auditory thalamus acting as a relay between the inferior colliculus and the auditory cortex. In this study, the volume of the right MGN was significantly decreased in the LHON patients compared to the healthy control subjects.

Another important result of this study is the correlation between the right RNFL averaged thickness and the volume of the right LGN in the LHON group. Changes within the RNFL structure are one of the most common ophthalmological examination results in LHON patients [35,36,37]. A recent study also showed a unique process undergoing thickening to thinning in the patients with LHON [38]. A study with the application of diffusion MRI to the quantitative assessment of the optic nerve in LHON showed significant correlation of the optic tract fractional anisotropy value and the thickness of the RNFL [39]. Nevertheless, in accordance with the results of Hedges et al. [40], RNFL averaged thickness changes with the prolongation of the disease, even within weeks; thus, the correlation with the decreased volume of the LGN on the same lateral side can be evidence that, along with the changes in RNFL, LGN volume decreases.

The analysis of the selected dimensions of the optic nerves showed no differences between the right and the left optic nerve in the LHON participants. This observation is different from that presented in our previous paper [25]; however, in comparison to the previous findings, the group presented in this study was not composed of patients treated with idebenone, which may be the reason for the lack of intra-group differences. Nevertheless, we found a significant correlation between the volume of the right LGN and the right optic nerve surface area in the first measurement point. The previous study showed that optic nerve atrophy can be associated with the process of RNFL thinning in the LHON participants [41]. The optic nerve atrophy process is also one of the most common findings reported in LHON subjects [6,42,43]. Nevertheless, the connection between the decreased LGN volume and the optic nerve surface area suggests that the changes in the optic tract of LHON can be connected with each other.

The limitation of this study was the relatively small group size (15 patients), which might at least partially reduce the statistical power of the main findings; however, the disease is very rare in the Polish population, especially among adults, and the scans were obtained from 7 Tesla MRI used for research only. Secondary, the patients did not undergo any functional assessment of the auditory system. Furthermore, to analyze the relationship between the volume loss of the LGN and MGN structures and the atrophy of the white matter fibers that form the visual pathway, a hybrid study combining submillimeter morphometry and the diffusion imaging should be done in LHON. The reason for the third important limitation of this study, which is a lack of additional examination of ophthalmic parameters of our patients (i.e., vision acuity or the field of view), is acquiring the information from the initial interview, during which the patients described themselves as functionally blind. Future studies should also analyze the potential impact of idebenone therapy on the changes in the optic nerve dimensions or LGN/MGN volumes. Lastly, we have not used T2 data to improve pial surfaces during the segmentation protocol in Freesurfer software; nevertheless, the pial surface errors were manually examined and corrected by a neurologist during the analysis.

## 5. Conclusions

In conclusion, this is the first in vivo 7 Tesla study that examines changes in the volume of LGN and MGN in LHON participants. The ultra-high-field magnetic resonance imaging allowed quality visualization and analysis of the LGN and MGN, serving as a powerful in vivo diagnostic tool in the diagnostic process and the evaluation of the course of LHON disease. A comparative analysis between the controls and LHON patients revealed a decreased volume in the right LGN and the right MGN area. Furthermore, the volume of the right LGN was significantly correlated with the averaged thickness of the right RNFL and the right optic nerve surface area located proximally to the eyeball in the LHON participants.

## Figures and Tables

**Table 1:** Demographic and clinical data of research groups.

	LHON (*n* = 15) M (SD)	HC (*n* = 15) M (SD)	*t* Value or χ^2^	*p*
Age (years)	36.21 (14.41)	32.53 (7.42)	0.02	0.98
Education (years)	15.33 (1.98)	16 (1.55)	−1.82	0.67
Sex (% male)	86	66	1.67	0.19
Duration of illness (months)	132 (144.32)			
Mitochondrial mutation 11778G > A (%)	100			
RNFL averaged thickens (left)	62.153 (2.81)			
RNFL averaged thickens (right)	62.054 (3.15)			

Note. M—mean; SD—standard derivation; χ^2^—chi-square distribution; RNFL—retinal nerve fiber layer; *t*—Student’s *t*-test.

**Table 2:** Mean dimension values of the left and the right optic nerves measured at three different points for the LHON (*n* = 15) participants.

	Right M (SD)	Left M (SD)	Z	*p*
Length (cm)	4.18 (0.32)	4.23 (0.23)	−0.22	0.78
Diameter (mm) 1	3.52 (0.56)	3.75 (0.77)	−0.72	0.41
Diameter 2	2.28 (0.5)	2.27 (0.45)	0.07	0.91
Diameter 3	3.20 (0.22)	3.22 (0.29)	−0.12	0.86
Surface area (mm^2^) 1	12.93 (3.34)	13.11 (4.73)	−0.88	0.22
Surface area 2	3.81 (1.14)	3.77 (1.31)	0.22	0.61
Surface area 3	8.12 (1.22)	8.15 (1.34)	−0.18	0.69

M—median; SD—standard deviation; Z—z-score.
